# An evaluation of the Australian Community Pharmacy Agreement from a public policy perspective: industry policy cloaked as health policy?

**DOI:** 10.1186/s40545-023-00571-y

**Published:** 2023-06-12

**Authors:** John K. Jackson, Shane L. Scahill, Michael Mintrom, Carl M. Kirkpatrick

**Affiliations:** 1 Centre for Medicine Use and Safety (CMUS), Faculty of Pharmacy and Pharmaceutical Sciences, Monash University, 381 Royal Parade, Parkville, VIC 3052 Australia; 2 School of Pharmacy, University of Auckland, Auckland, New Zealand; 3 School of Social Sciences, Faculty of Arts, Monash University, Parkville, VIC Australia

**Keywords:** Policy theory, Community pharmacy, Pharmacy practice, Medicines access

## Abstract

**Background:**

A series of Community Pharmacy Agreements (Agreements) between the Federal government and a pharmacy-owners’ body, the Pharmacy Guild of Australia (PGA) have been influential policy in Australian community pharmacy (CP) since 1990. While ostensibly to support the public’s access and use of medicines, the core elements of the Agreements have been remuneration for dispensing and rules that limit the establishment of new pharmacies. Criticism has focused on the self-interest of pharmacy owners, the exclusion of other pharmacy stakeholders from the Agreement negotiations, the lack of transparency, and the impact on competition. The objective of this paper is to determine the true nature of the policy by examining the evolution of the CPA from a policy theory perspective.

**Methods:**

A qualitative evaluation of all seven Agreement documents and their impact was undertaken using policy theories including a linear policy development model, Multiple Streams Framework, Incremental Theory, the Advocacy Coalition Framework, the Theory of Economic Regulation, the Punctuated Equilibrium Framework, and Elite Theory. The Agreements were evaluated using four lenses: their objectives, evidentiary base, stakeholders and beneficiaries.

**Results:**

The PGA has acted as an elite organisation with long-standing influence on the policy’s development and implementation. Notable has been the failure of other pharmacy stakeholders to establish broad-based advocacy coalitions in order to influence the Agreements. The incremental changes negotiated every 5 years to the core elements of the Agreements have supported the publics’ access to medication, provided stability for the government, and security for existing pharmacy owners. Their impact on the evolution of pharmacists’ scope of practice and through that, on the public’s safe and appropriate use of medication, has been less clear.

**Conclusions:**

The Agreements can be characterised predominantly as industry policy benefiting pharmacy owners, rather than health policy. An emerging issue is whether incremental change will continue to be an adequate policy response to the social, political, and technological changes that are affecting health care, or whether policy disruption is likely to arise.

## Introduction

In Australia, 97% of the community pharmacy (CP) sector is pharmacist-owned, small businesses (pharmacies) delivering primary healthcare services within a commercial retailing environment [1]. The sector has a wide range of stakeholders and a complex policy environment covering amongst other matters, practitioner registration and practise, pharmacy ownership and location, and medicines quality, access, dispensing and funding [2]. State and Territory governments have principal legislative responsibility for pharmacists’ practise and premises however a Federal government policy, the Community Pharmacy Agreement (CPA or Agreement) is arguably equally as influential [3, 4].

The term CPA encapsulates 7 consecutive five-year agreements (1990–2025) negotiated between the Federal governments and the Pharmacy Guild of Australia (PGA), an industrial body representing pharmacy owners [5]. The Agreements have controlled pharmacy remuneration for dispensing prescriptions for medicines listed on the schedule of the Pharmaceutical Benefits Scheme (PBS), the national pharmaceutical insurance program [6]. The Federal government expenditure on pharmaceutical benefits (and related services) rose from $1.26B in 1989–1990 to $16.4B in 2021–2022, and now accounts for approximately 15% of its total health expenditure [7, 8]. Of this, the current Agreement projects $2.6B will be paid annually to the owners of Australia’s approximately 5900 pharmacies for PBS dispensing and services. A further $1.9B will be received by them as patient co-payments for prescriptions [9, 10]. Dispensing revenue forms the largest portion of CP revenue (68.5%) and underpins the viability of most pharmacies [11].

The initial Agreement was negotiated to address a conflict between the government and the profession in the late 1980s [12]. A ‘market failure’ arose when the Federal government sought to reduce the wholesale margin on PBS-listed medicines and payments to pharmacies for PBS dispensing, and decrease the number of approved pharmacies, resulting in conflict with the profession [13]. The Remuneration Tribunal cut the dispensing fee by 23%, and in the hostility that developed, pharmacists took the exceptional action of holding public rallies with some radical owners ‘striking’ (closing their pharmacies) [14, 15]. A militant Community and Pharmacy Support group emerged and challenged the PGA’s approach to the negotiations. Internal differences arose within both the PGA leadership and the government and the Prime Minister became involved in the resolution. A “micro-economic reform package” submitted by the PGA to the government resulted in a 5-year Agreement which resolved the dispute [16, 17].

In addition to setting remuneration for wholesaling and dispensing of medicines on the PBS, the initial Agreement (known as the Guild–Government Agreement until the term CPA was used for the third Agreement) forced structural reform of the CP sector through funded closure and amalgamation of pharmacies [17]. Controls on the location of pharmacies approved to dispense PBS prescriptions (location rules) were introduced to ensure access to PBS medicines via ‘commercially viable, competitive, responsive and geographically distributed pharmacies’ [18]. All 7 Agreements, negotiated by both progressive and conservative governments over 30 years, have included the core elements of dispensing remuneration and location rules. Practice-based research was funded from the 2nd to the 6th CPA, and funding non-dispensing professional programs (Programs) commenced in the 3rd CPA [19].

The CPA has become an institution and most community pharmacists have known no other policy framework. In contrast, funding arrangements negotiated between the Federal government and other health professions have not been sustained (Pathology Funding Agreement 1996–2016, Diagnostic Imaging Agreement 1998–2008, and Memorandum of Understanding with organisations representing general practitioners 1999–2002) [20].

By the time of the 3rd CPA, it was seen as ‘the determinant of sustainable practice for the profession’ [4]. The CPA has provided stability for the Federal government and a high level of security for existing pharmacy proprietors, however most CP stakeholders have not been directly engaged in Agreement negotiations. These include consumers, State and Territory governments, pharmacy and non-pharmacy regulators, pharmaceutical manufacturers, wholesalers, and pharmacy organisations other than the PGA (a professional body, the Pharmaceutical Society of Australia (PSA) is signatory to a small section of the 7th CPA). Criticism has been raised by some stakeholders regarding the nature, administration and outcome of the Agreements [21–25]. Critics have focused on their exclusion from Agreement negotiations, the CPAs’ anti-competitive nature, the advantages accruing to existing proprietors, and the lack of transparency particularly in relation to the funding of Programs.

In addition to significant population, prescription volume and PBS expenditure growth, Australia has experienced notable social, political, technological and economic change since 1990 [26]. Examples of changes in health care which have yet to be incorporated into the CPA include collaborative patient-focused models of care and funding models aimed at enhanced value and quality [27–30].

Policies are intentional, expedient choices made by authoritative agents within given circumstances to achieve stated objectives [31]. Governments make public policies with the objective of addressing specific existing or potential problems. The idealistic view of public policies is that they are developed with the engagement of the range of stakeholders that they are likely to affect, for the benefit or protection of needy sectors of the public [31, 32]. Due to governments’ regulatory and funding roles, public policies can be particularly impactful. However, as they evolve within a political ethos, the political nature of their development may result in the policies supporting private interests over public [33]. In the extreme, policy capture occurs “*where public decisions over policies are consistently or repeatedly directed away from the public interest towards a specific interest*” [34].

As the element of Australia’s universal health coverage that supports the public’s access to essential medicines through significant government and public expenditure, the CPA is perceived principally as Federal government health policy. While some studies have described the CPA, no prior studies have analysed their true nature as policy [20, 35]. The objective of this paper is to examine and characterise the CPA and examine its past and likely future evolution from a policy theory perspective.

## Methods

This paper is novel and makes a significant contribution by applying a range of policy theories to this dominant pharmacy sector public policy. To commence the process, a linear policy development model incorporating stages of problem definition, agenda, policy formulation, implementation, and evaluation, was used to present the evolution of the original Agreement [36, 37]. However, this linear process assumes simplicity and fails to explain the multi-faceted aspects of policy development and the complexities that arose over the series of Agreements. We subsequently applied a range of theoretical frameworks relating to specific aspects of policy development in a qualitative, realist evaluation of the seven Agreements. Our aim was to determine how the Agreements evolved, describe their underlying architecture, and identify for whom they work [19, 38]. The policy theories include the Multiple Streams Framework, Incremental Theory, Advocacy Coalition Framework, Theory of Economic Regulation, Punctuated Equilibrium Framework, and Elite Theory (Table 1) [31, 33, 39–44]. Four lenses, namely the stated objectives of the Agreements, their evidentiary base, stakeholder engagement, and beneficiaries, were considered in the application of the policy theories. The 7 Agreement documents were analysed to assess the policy over the 30-year period and to determine the validity of the prevailing paradigm that the CPA is principally health policy.

**Table 1:** Policy theories applied in the evaluation of the CPA

Theory	Description
Multiple Streams Framework [37]	A crisis may be resolved if a window of opportunity arises by the 3 factors (or streams) of problem, politics and policy each evolveing to a stage of coalescence
Incremental Theory [38]	Only minor changes are made on review as policy makers assume that the existing policy is legitimate and the overall outcomes are adequate
Advocacy Coalition Framework [39]	Parties would be expected to overcome potentially conflicting and disparate objectives and interests to focus on their common interest in a policy
Theory of Economic Regulation [40]	The political nature of policy development may result in it benefiting private interests over its less influential intended beneficiaries
Punctuated Equilibrium Framework [41]	Public policies are characterised by long periods of stability punctuated by brief episodes of dramatic change
Elite Theory [42]	The self-interested behaviour of agencies that capture policy development

## Results

The linear policy development model representation of the evolution of the original Agreement is shown in Table 2.

**Table 2:** Development of the first agreement in a linear policy development model

Stage	Description
Problem definition	A conflict existed in which community pharmacist sought to maintain viability while the government sought to reduce the cost of the PBS
Agenda	A proposal for micro-economic reform including pharmacy closures and a formula for dispensing remuneration was submitted by the PGA
Policy adoption	The proposal was adopted by the government and received parliamentary approval under the National Health Act (1953)
Policy implementation	An Authority was established to regulate pharmacy approvals. The Government managed other aspects of the agreement with PGA input
Policy evaluation	No publicly available evaluation was undertaken of the first agreement

### The stated objectives of the CPA

The General Objectives clause of the 1st CPA included the intention to “*produce a more efficient community pharmacy structure in Australia resulting in benefits to both parties*”. This focus on the pharmacy network rather than patients was perpetuated in the 2nd CPA objectives to “*maintain the benefits of restructuring*…” and “*not to provide for an increase in the number of approved pharmacies*…” [17, 19]. The 3rd CPA included 6 ‘Principles’ and 10 ‘Objectives’. The first principle was ‘*providing consumers with reasonable equality of access to quality pharmacy services in their local community*’ [45]. Pharmacy services are not defined in the Agreement, however this aim can be aligned with one of the objectives of the National Medicine Policy 2000 (NMP) which was published contemporaneously, namely *‘timely access to the medicines that Australians need, at a cost* (that) *individuals and the community can afford’* [46]. The second principle was*’ ensuring that consumers receive quality patient care and outcomes*’. Quality patient care is not defined in the Agreement, however this aim can be aligned with a second objective of the NMP; the *‘quality use of medicine’* (QUM, i.e. safe and appropriate use).

Another 3rd CPA principle refers to ‘*extending the cooperative approach evident in the first two Agreements between the Guild and the Commonwealth*’. The PGA remained the sole negotiator with the Federal government until the PSA became signatory to a section of the 7th CPA. That section identifies the PSA as custodian of the profession’s code of ethics, competency framework, practice standards and guidelines and commits the PSA to support the design and evaluation of Programs [9].

The 3rd CPA objectives refer to *‘a network of well distributed, accessible, and viable community pharmacies*’, ‘*fostering of a stable and viable community pharmacy sector’* and ‘*providing greater financial stability for the parties*’. These objectives address the government’s need for a network of viable community pharmacies as a means of ensuring public access to PBS medicines, but also support the commercial interests of PGA members, who are all community pharmacy owners.

In addition to further objectives focussed on services to rural, remote and indigenous communities, and the application of information technology, the 3rd CPA objectives included a ‘*continued development of an effective, efficient and well-distributed community pharmacy service in Australia which takes account of the recommendations of the Competition Policy Review of Pharmacy and the objectives of National Competition Policy*’. How the recommendations were to be addressed was not specified in the Agreement.

Objectives aimed at access, quality, a stable environment and cooperation, are included in the 4th CPA and 5th CPA along with objectives of being patient-focused, evidence-based (in relation to Programs), accountable, efficient, effective, transparent, and sustainable. No specific objectives statement was included in the 7th CPA however a Community Pharmacy Consultation Committee (CPCC) was proposed “*to support the achievement of the Commonwealth and the Guild’s objectives under the 7CPA*” [9].

The objectives specified in the Agreements are classified in Table 3 using Hancock’s criteria for evaluating the adequacy of health policy, plus other criteria [47].

**Table 3:** Principles and objectives of the CPAs classified according to Hancock’s criteria for evaluating the adequacy of health policy, plus other criteria

The objectives of the CPA						
1st CPA 1990-1995	2nd CPA 1995-2000	3rd CPA 2000–2005	4th CPA 2005–2010	5th CPA 2010–2015	6th CPA^a^ 2015–2020	7th CPA^a^ 2020–2025
1. Access and affordability^b^						
		Consumer access to quality ph’cy services Expansion of prof. roles including medicine reviews	Location rules ensure access to PBS medicines and benefit Aust consumers	Patient-focused care All have access to the PBS	Access to new PBS medicines	Access to patient-focused programs
2. Effective healthcare treatment and care^b^ (outcomes)						
		Co-ordinated multi-disciplinary services	Optimise effectiveness and value. Efficient and effective outcomes Improve h/care outcomes via evidence-based professional programs	Health benefits of evidence-based programs		
3. Longer-term sustainability^b^ (incl. viability)						
		Stable, predictable environment Network of well-distributed, viable and accessible CPs Financial stability for C’wth and PGA	Fair price paid to pharmacists by Commonwealth for PBS	Fair price paid to pharmacists by Commonwealth for PBS Sustainable PBS	Cost-effective and sustainable PBS	
4. Economic efficiency^b^						
		Well developed, effective, efficient and well-distributed CP network	Funds properly expended—efficient and accountable Maximise value to taxpayers Effective and efficient CP network Improved efficiency through competition between ph’cies	Efficient PBS Efficiency through competition		
5. Quality of care^b^						
		Quality patient care outcomes Quality personalised ph’cy service Intro. of qual. standards	Quality pharmacy service to consumers		Quality Use of Medicines (QUM)	Support for QUM
6. Public interest accountability^b^ (and transparency)						
			Transparent and accountable in exp of funds Accountability efficiency and transparency in admin and delivery Meets standards of accountability	Accountability and transparency in admin and delivery of programs Proper expenditure of funds		Transparent and appropriate out-of-pocket costs
7. Democratic participation and openness of decision-making^b^						
						Involvement of a broader more inclusive set of stakeholders
8. Social equity and social justice^b^						
	Maintain services in remote and isolated areas	Access for rural and remote, and Indigenous people	Programs target areas of need Improvements for indigenous people	Target areas of need incl. indigenous people		
9. Other criteria (not Hancock)						
A more efficient CP structure … benefit to both parties	Maintain benefit of restructuring Not increase the number of pharmacies	Extended cooperation evident in CPA 1 and 2	Co-operative relationship between C’wth and PGA	Co-operative relationship between C’wth and PGA		Cooperation between signatories and a broader set of stakeholders
		Stable and viable CP sector CP services take account of Competition Policy IT improved med management	Stable and viable CP sector. Location rules ensure commercially viable and sustainable network and increase flexibility to respond to need	Sustainable and viable CP sector, flexible to respond to needs. Clear roles for C’wth and PGA		Predictable remuneration to support CP network

Code: ^a^6CPA and 7CPA had no specific objectives. 7CPA listed objectives are commitments in the Agreement

^b^Hancock’s criteria

ATSI: Aboriginal and Torres Strait Islander, CP: community pharmacy, Ph’cy (‘cies): Pharmacy(ies), C’wth: Commonwealth/Federal government ara>

### The evidentiary base of the CPA

Evidence-informed policy is an aspirational concept that assumes policy evolves through a logical process of formulation, implementation, evaluation and review, based on systematic research and valid data [48, 49]. It creates an impression of certainty derived through a rational approach and systematic research, when in fact policy is likely to be an expedient compromise influenced by uncertainty and ambiguity [50].

The initial Agreement arose through exceptional circumstance. A window of opportunity arose when, in accord with Kingdon’s Multiple Streams Framework, the three factors of problem, politics and policy each evolved to a stage of coalescence. This enabled the crisis that existed between the government and pharmacy owners to be resolved [39].

Lindblom’s Incremental Theory states that policy makers act on the assumption that the existing policy is legitimate and the overall outcomes are adequate [40]. Consequently, at times of a policy review, the parties are only open to adjustment to limited aspects of the policy under consideration, and any changes are consequently minor. Subsequent CPA negotiation conducted in private by the same two parties, perpetuated the key elements of the 1st CPA, usually with marginal adjustments to rates, fees, formulas and Programs. Incremental adjustments minimise uncertainty in relation to outcomes and are likely to achieve workable outcomes rather than optimal outcomes.

In the absence of an initial evidentiary base and with re-negotiation resulting in incremental change, external reviews become important for policy validation. In 2011, notice was given in the Australian Senate for a review of the 5th CPA by the Community Affairs Reference Committee. The review did not proceed, but the proposed matters to be reviewed illustrate the scope of concern with the CPA at the time. The matters included:

- pharmacy remuneration and value for taxpayer funds;
- the effectiveness of governance arrangements;
- the Pharmacy Location Rules;
- the CPA process involving a single entity, the PGA;
- potential conflicts of interest between the provision of ethical and professional pharmacy services and the commercial interests of pharmacy owners [53].

A small number of official reviews have assessed various aspects of the CPA however adoption of their recommendations in subsequent CPAs has been minimal [54–56]. The Review of Pharmacy Remuneration and Regulation, which was a requirement of the 5th CPA and attracted 510 submissions, made 45 recommendations of which the government accepted just 4 [56, 57]. Recommendations in the following areas (all relevant to the CPA) were not accepted by the government: pharmacy ownership, automation, minimum services, remuneration, location rules, harmonising legislation, and the scope, negotiation and principles of the CPA.

Federal agencies such as the Productivity Commission and the Australian Competition Commission have made recommendations regarding the CPA including the anti-competitive nature of the location rules. The National Competition Policy Review of Pharmacy Legislation stated the rules restrict free and effective competition and the Review ‘*could not show that the restrictions are entirely in the public interest’* [54]. In spite of the 3rd CPA explicitly stating that recommendations of National Competition Policy must be considered, the location rules have been retained in subsequent Agreements, albeit with incremental modifications [22, 56].

A 2014 Victorian Parliamentary Inquiry into Community Pharmacy made 17 recommendations, 10 involving the Commonwealth of which 3 referred specifically to the 6th CPA, none of which were adopted in subsequent Agreements [58].

In 2015 the Australian National Audit Office (ANAO) inquiry into the Administration of the 5th CPA *‘identified scope for improvement in key aspects of the (*Federal Health*) department’s general administration which covered the 5CPA’s development, negotiation and implementation phases’* [55]. The ANAO determined the CPA failed to deliver on government goals, that only a small portion of the money allocated to patient-focused programs was spent on direct patient care, dispensing fee remuneration received by pharmacists was inflated due to the use of an indexation rate higher than applied by the Department of Finance, and money paid to the PGA to administer programs came from budgets allocated for payment of pharmacists for professional services. The ANAO questioned the supervision of the PGA’s management of CPA funds, as *‘the department did not assess whether financial framework requirements would apply to the Pharmacy Guild officials when making payments of public money pursuant to the administration of 5CPA professional programs’* [55]. These findings were in spite of the Objectives of Part 4 of the 5th CPA making specific reference to the proper expenditure of funds, and accountability and transparency in the administration and delivery of programs in accordance with Commonwealth guidelines [59].

### Stakeholder engagement in the CPA

It is sound public policy development for agencies and interested parties likely to be affected by a policy to be engaged in the development process. Sabatier argues that parties with varied and potentially conflicting objectives would be expected to overcome their disparate interests to form Advocacy Coalitions focused on their common interest. In relation to the CPA, groups of non-government CP stakeholders critical of the CPA, would be expected to collaborate to influence Agreement negotiations [41, 61, 62]. In spite of this expectation, patient and other community groups, State and Territory governments, professional and industrial bodies, academics, and commercial competitors of CP have failed to form coalitions with agreed positions in relation to the CPA. State and Territory governments have primary legislative responsibility for pharmacy, and their relations with the Federal government regarding health financing is a priority policy area, yet they have failed to act in coalition with respect to the Agreements [58, 63].

The Australian Pharmacy (Liaison) Leaders Forum (APLF) claims to be ‘a coalition of representatives from pharmacy organisations who work together on issues of national importance to the pharmacy profession and the public’. Consisting of executive members of major pharmacy organisations (including the PGA), the APLF may have been expected to form a multi-party position on the CPA, but has failed to do so [64]. The Pharmacist Coalition for Health Reform was a second opportunity for an advocacy coalition within pharmacy. Established in 2011 to promote the interests of individual pharmacists, it consisted of 2 professional bodies (the PSA and the Society of Hospital Pharmacists of Australia), a pharmacists’ union (Professional Pharmacists Australia (then APESMA)), and a student body (National Australian Pharmacy Students Association). This coalition was disbanded within 18 months due to differing opinions with no notable impact on the CPA [65].

No pharmacy organisation has developed a long-standing alliance with patient representative bodies such as the CHF in relation to the CPA, and stakeholders such as pharmaceutical manufacturers and wholesalers have not publicly engaged in advocacy coalitions.

The Federal government consulted with stakeholders prior to the negotiation of the 7th CPA and the PGA, PSA, CHF and National Aboriginal Community Controlled Health Organisations (NACCHO) have been engaged as members of the CPCC. On assessment of the implementation and operation of the Agreement the PGA was in complete agreement with all 12 criteria however PSA, CHF and NACCHO did not agree or were unsure of more than half of the criteria [10].

### The beneficiaries of the CPA

Stigler’s Theory of Economic Regulation argues that public policy is theoretically for the benefit of needy sectors of the community who frequently have little influence in the political arena. However, influential stakeholders can gain undue benefit, particularly when a policy grants licences with associated revenue streams. Holders of licences can form self-interested ‘elites’ [66]. The PGA has demonstrated most of the attributes of an elite within Agreement negotiations, and through this status its members have been major beneficiaries of the CPA [67, 68].

Elite Theory states elites frequently hold monopolistic positions, demonstrate a high level of acumen in relation to the political and bureaucratic processes of policy development, and engage with ‘proximate policy makers’ such as legislators and bureaucrats [43]. Elites normally support the status quo that exists due to their prior influence on policy and consequently are inclined towards incremental rather than major change.

An elite may seek more than a revenue stream. Stigler argues that industry groups that use their power to influence public policy seek to ‘control entry of new rivals’ [33]. CPA location rules which have been a hallmark of every CPA, have theoretically been maintained to ensure appropriate distribution of pharmacies. By limiting opportunities to establish new pharmacies, the rules restrict entry of rival proprietors, thereby benefiting PGA members as existing pharmacy owners. As a consequence of funded closures in the 1st CPA and on-going location rules, there are currently fewer pharmacies in Australia than in 1990 and beneficially to proprietors, the population to pharmacy ratio has increased from approximately 3000 per pharmacy to over 4400 [73].

## Discussion

This paper aims to examine the CPA from a policy perspective using four lenses: the Agreements’ stated objectives, the evidentiary base, stakeholders and beneficiaries. The prevailing paradigm that the CPA is public health policy is reinforced by two 3rd CPA principles of providing ‘*access to quality pharmacy services’* and ensuring ‘*quality patient care and outcomes’.* In addition to this health focus, Table 3 indicates the Agreements included an industry focus of sustaining a viable, sustainable and efficient community pharmacy sector, an economic focus of appropriate expenditure of funds, and a governance focus of efficiency, accountability and transparency.

In addition to having divergent objectives, it is apparent that the development of the original CPA was circumstantial and politically expedient, rather than evidence-based. Chiv et al. argue that policy governing pharmacists’ practice should be evidence-informed and the CHF states specifically that the CPA should be evidence-based [51, 52]. In spite of the limited evidence, there has been no over-arching review of the intent of the CPA, and the reviews that have occurred have had limited impact. While the NMP was comprehensively reviewed after 22 years, no major reform of the CPA has occurred after 30 years [60].

One stakeholder, the PGA has achieved exceptional status in relation to the CPA. The initial CPA was a major disruptive change in Federal policy in 1990. Tuohy argues that major changes in policy that arise through exceptional circumstances frequently result in exclusive relationships between the participating parties being maintained for extended durations [13]. Arising from its participation in the initial Agreement and in the face of widespread criticism, the PGA has maintained an exceptional role in all subsequent CPA negotiations. In spite of their expressed concerns, other CP stakeholders have failed to collaborate to achieve critical mass in their political influence so as to challenge the role of the PGA in Agreement negotiations and achieve outcomes more favourable to their interests. The self-interest of the PGA in seeking to benefit its members through the CPA process is to be expected, however the tolerance of the Federal government, and the absence of effective engagement by other stakeholders, are less understandable.

If the CPA is principally public health policy, the public can expect to be the main beneficiaries. While negotiating the first agreement created the opportunity for the PGA to become the exclusive negotiating party for subsequent Agreements, its retention of this status has been enabled by successive Federal governments which have legislated to negotiate just *‘with the agency representing the majority of pharmacy proprietors’* [10]. In adopting this criterion, governments enabled the PGA to demonstrate the prowess of an elite, and for negotiations to result in incremental changes to the prior agreements while maintaining benefits for existing pharmacy owners. They have facilitated the CPA maintaining a focus on dispensing remuneration and proprietorial interests, rather than on wider public health matters that benefit consumers.

The PGA is described as a major lobby group within Australia and in the lead up to the 2019 and 2022 Federal elections made substantial political donations [67, 69, 70]. The PGA has maintained its negotiating role without public confirmation of its ‘majority representation’ status, and governments have maintained their position in spite of members of the pharmacy group with the largest turnover, the My Chemist/Chemist Warehouse banner group not holding PGA membership, and over 20% of the PBS now being expended through hospital pharmacies [11, 71].

Alternate arrangements could deliver better beneficiary and stakeholder equity. The PGA’s exclusive status in the CPA is in contrast to the Pharmaceutical Services Negotiating Committee (PSNC) representing pharmacy contractors in England. The PSNC consists of both individual pharmacy owners elected regionally and nominees of pharmacy owner organisations [72].

The Council of Australian Governments (COAG), a forum that included the Prime Minister, State and Territory Premiers and Chief Ministers (1992 to 2020), and which commissioned the National Competition Review of Pharmacy, was a platform that could have pushed for a more equitable, national approach to the CPA [74]. The subsequent Health Ministers Meetings which consider national strategies for a range of health services (maternity, palliative care, chronic conditions) is another potential pathway for the State and Territory governments to influence the CPA [75].

Arising from this analysis, three aspects of the CPA as public policy warrant consideration: has the CPA met its public health objectives, what is the true policy nature of the CPA, and what is likely to be its future?

### Public health and the CPA

The public’s notional interest in the CPA includes being able to have timely and affordable access to medicines and to be supported in the safe and appropriate use of medicines. Of the $16.4B allocated by the government to the 7th CPA just $1.2B is for Programs. With the bulk of the funding ($15.2B) applied to dispensing and supporting the existence of the CP network, the CPA has contributed strongly to the first objective, however its impact on the appropriate use of medicines through the development of ‘quality pharmacy services’ and Programs, remains unassessed.

QUM is central to pharmacists’ roles as medicines experts and CPA funding of non-dispensing Programs is intended to support the enhanced scope and quality of pharmacist’s practice. Research to support new Programs and the profession’s aspirations for an expanded scope of practice including remunerated patient-focused roles, align with the public health objective of QUM [76, 77]. Over five CPA’s, approximately $95 million was committed to research to ‘improve clinical outcomes for consumers and/or extend the role of pharmacists in delivery of primary healthcare services’ [78]. This research led to the introduction of a small number of funded Programs, some of which have not been sustained (e.g. Clinical Interventions) [5]. Between the 3rd and 6th CPAs, Programs received between 1.29% and 2.77% of CPA funding. The impact of the Programs was reported as small in 2015 and uptake reported as limited by 2020 [78, 79]. In the first year of the 7th CPA, projected funding covered just three clinically focused Program areas (Medication adherence programs $105.5 M, Medication Management Programs $96.4 M, Indigenous peoples Programs $12.6 M) [9]. Services that pharmacists are funded to deliver in other countries but not in Australia include ordering laboratory tests, screening patients, treating minor ailments, and implementing chronic care plans prepared by medical practitioners [80].

Not only have the governments not specified in the CPAs the minimum professional services expected from CPs, the Agreements appear to have had limited impact in this area as Program development has been constrained and price- and volume-focused pharmacies have emerged at the expense of professional service-focused pharmacies [56, 78].

### The true policy nature of the CPA

The CPA reflects the dichotomy in CP of healthcare provision and small business viability. The first Agreement, appropriately called a Guild–Government Agreement, was a response to a market failure arising from a funding dispute. It was negotiated by an industrial organisation to regulate supply-linked funding, delivered micro-economic reform of the sector through funding of pharmacy closures, and established controls that limited market entry, all of which point to it being industry policy. Subsequent Agreements which included objectives of ‘consumer access to quality pharmacy services’ and ‘expansion of professional roles including medication reviews’ and included funding for research and Programs, were reframed as health policy and renamed Community Pharmacy Agreements. However, one consistent objective of all 7 Agreements has been the viability of community pharmacies.

The CPA is in effect industry policy cloaked as health policy. It is an example of the Federal government’s use of its funding powers to control the health sector in the absence of constitutional responsibility for regulation and delivery of health care [81].

### The future for CPA as public policy

Policy change is normally slow, particularly when an elite actor seeks to maintain the status quo [82]. Policy developed in this manner reflects the past more than the future, does not support innovation and will likely not keep up with societal change. This gridlock is likely to be punctured by a significant disruptive policy reset as a result of critical evaluation stimulated by concern with the effectiveness or cost of a policy program, or some form of crisis [83, 42].

The CPA may have been an appropriate industry policy reset at the time of the market failure of the late 1980s, however incremental change has failed to accommodate developments in the Australian health care system such as collaborative care models, quasi-corporatisation of pharmacy businesses and value-based health funding. While a crisis is not yet apparent, an overt policy failure may arise if changes in the CPA do not reflect social, political, technological and economic developments, and the practice of CP diverges from the policy. Once the status quo is no longer tolerable, the need to address the problem becomes a political imperative, not dissimilar to the theory of Punctuated Equilibrium in the face of policy gridlock [42]. Looking to the future, the CPA’s existing breadth and scope will not accommodate converging developments in artificial intelligence, biotechnologies, robotics, enabled by the Internet-of-Things [84]. If contemporary health care concepts and emerging technologies are not reflected in future negotiations, the risk is that the policy framework will face disruptive change [29, 30].

### Limitations and future work

This study was limited to an Australian pharmacy-related policy, however we believe the approach has application in other disciplines and countries. Other policy theories exist. We have applied those that we believe are most relevant to the CPA. Arising from this study, further research into stakeholder perceptions of the CPA and its impact on pharmacy services and the pharmacy profession is warranted.

## Conclusion

This paper set out to utilise policy theory to assess the CPA as a public policy. The policy has provided stability for the government although issues have been raised regarding efficiency and accountability. It has helped assure the public access to medicines but to a much lesser extent, supported QUM through the development of pharmacists’ practice. If health policy, the benefits of the CPA would flow to patients, however, existing pharmacy owners gain a unique advantage, the costs of which are diffused throughout the healthcare system. Without greater transparency in the negotiations, it is not possible to determine the extent to which the PGA has captured the CPA, but it leaves the appearance of the PGA as an elite and the CPA as industry policy cloaked as health policy.

The limitations of the CPA as a policy can be attributed to the origins of the initial Agreement as a solution to a crisis, the competence of those responsible for crafting and managing subsequent Agreements, the restrained behaviour of excluded stakeholders, and the self-serving focus and influence of the PGA. The status of the PGA as sole negotiator with the government must be contrasted with the failure of advocacy by other CP stakeholders which can be interpreted as a reflection of their limited political acumen vis-à-vis the PGA.

The stability that has existed and sustainability of the CPA is likely to be challenged by the lack of consideration of the interests of the broad range of CP stakeholders, specifically consumers, and by how well the policy adapts to and aligns with future public expectations and needs. Incremental change has been a feature of CPA negotiations, however changes within health care may necessitate a shift in the government’s approach to the policy at the conclusion of the current Agreement.

## Data Availability

All documents used in the research are in the public domain.

## Abbreviations

Agreement: Community Pharmacy Agreement
ANAO: Australian National Audit Office
APLF: Australian Pharmacy Leaders Forum
CHF: Consumers Health Forum of Australia
COAG: Council of Australian Governments
CP: Community pharmacy
CPCC: Community Pharmacy Consultation Committee
CPA: Community Pharmacy Agreement
NACCHO: National Aboriginal Community Controlled Health Organisations
NMP: National Medicines Policy
PBS: Pharmaceutical Benefits Scheme
PGA: Pharmacy Guild of Australia
PSA: Pharmaceutical Society of Australia
QUM: Quality Use of Medicines
